# Patterns of Health Service Use Among Young People With Cerebral Palsy in England

**DOI:** 10.3389/fneur.2021.659031

**Published:** 2021-05-12

**Authors:** Jennifer M. Ryan, Grace Lavelle, Nicola Theis, Cherry Kilbride, Marika Noorkoiv

**Affiliations:** ^1^Department of Public Health and Epidemiology, Royal College of Surgeons in Ireland (RCSI) University of Medicine and Health Sciences, Dublin, Ireland; ^2^College of Health, Medicine and Life Sciences, Brunel University London, London, United Kingdom; ^3^Institute of Psychiatry, Psychology and Neuroscience, King's College London, London, United Kingdom; ^4^School of Sport and Exercise, University of Gloucestershire, Gloucester, United Kingdom

**Keywords:** cerebral palsy, health services, adolescent, rehabilitation, neurological disorders

## Abstract

**Background:** Although the provision of healthcare for people with cerebral palsy (CP) is typically focussed on childhood, many people with CP require access to services periodically throughout their life. Few studies have examined patterns of health service use among young people with CP in England. Understanding patterns of use may inform future service development.

**Objective:** To describe patterns of visits to rehabilitation and medical professionals among ambulatory young people with CP living in England, and identify factors associated with service use.

**Methods:** Sixty-two young people with CP aged 10–19 years [mean (SD) age 13.7 (2.5) years] in Gross Motor Function Classification System (GMFCS) levels I-III reported visits to a range of health professionals, hospital admissions and visits to the emergency department over a median duration of 34 weeks (min–max: 12–34 weeks). Negative binomial models were used to examine factors associated with number of visits.

**Results:** Physiotherapists were the most commonly used professional, with 67.7% of participants visiting a physiotherapist at least once, followed by dentists (66.1%), general practitioners (48.4%), occupational therapists (40.3%) and orthopaedic surgeons (40.3%). Physiotherapists were also the most frequently visited professional with a total of 473 visits (13.3 visits per person-year). Speech and language therapists (5.0 visits per person-year), occupational therapists (4.5 visits per person-year) and nurses (4.3 per person-year) were the next most frequently visited professionals. Age, GMFCS level, and speech impairment were associated with rate of visits to a physiotherapist.

**Conclusions:** The proportion of young people who visited medical and rehabilitation professionals during the study period varied considerably depending on the profession. Generally, the proportion of young people using services was low. In the context of limited resources, data on service use in combination with data on unmet need, may support the reorganisation of services to maximise benefits to young people with CP.

## Background

Cerebral palsy (CP) is a lifelong condition, with the majority of people with CP surviving to at least 60 years (1). Although impaired motor function is the core feature, people with CP can experience a range of associated impairments, such as epilepsy and intellectual disability (2). The type and severity of impairment experienced varies significantly between individuals. People with CP are intensive users of healthcare. Between 2000 and 2014, children and young people with CP in Northern Ireland accounted for 1.6% of total hospital admissions and 1.6% of total outpatient appointments, despite representing just 0.3% of the population (3). Although there is evidence that service use declines from childhood to adolescence and through to adulthood (4, 5), this may be because of difficulties accessing services rather than lack of need (6). Adults with CP have an increased risk of chronic physical health conditions, mental health conditions, and falls compared to adults without CP (7–10). Further, between 18 and 63% of young people with CP aged 14–18 years report needs in areas such as epilepsy, bone or joint problems, and control of movement, with between 10 and 45% reporting that their needs are not met (11).

Understanding patterns of health service use and predictors of service use is essential for planning service delivery. However, despite a significant proportion of young people with CP in the UK reporting healthcare needs (11), there is limited information available regarding service use in this group. A recent report found that, between 2004 and 2014, the rate of GP consultations was ~200 per 100 person-years among people with CP aged 10–14 years and 250 per 100 person-years among those aged 15–19 years in England (12). Rate of outpatient appointments were approximately 450 per 100 person-years among people aged 10–14 years and 350 per 100 person-years among those aged 15–24 years (12). Rate of inpatient visits were ~50 per 100 person-years among people aged 10–19 years (12). Another recent study of children and young people with CP aged 0–24 years in Northern Ireland reported that 68.4% had at least one hospital admission between 2004 and 2014 and 32.6% had at least one outpatient appointment between 2010 and 2014 (3). While providing important information using population based datasets, these studies do not describe the range of services used by young people with CP.

A survey of young people in Northern Ireland in 2008 identified the percentage of 12–18 year olds who visited a range of professionals during a 6-month period, which ranged from 3% for a psychologist to 92% for a physiotherapist (5). This study however was limited to non-ambulatory individuals with CP [i.e., those in Gross Motor Function Classification System (GMFCS) levels IV and V]. As people with CP in GMFCS levels IV and V typically have greater needs and are more frequent users of health services than people in levels I-III (4, 13–15), findings may not be applicable to ambulatory people with CP who constitute ~75% of the CP population (16).

While these studies provide essential information about service use among young people with CP, there remains gaps in our knowledge about the range of professionals used by young people with CP in the UK. In this paper, we aim to add to current knowledge by describing patterns of visits to rehabilitation and medical professionals among ambulatory young people with CP living in England, and identifying factors associated with service use.

## Methods

### Sample

Young people with CP who participated in a randomised controlled trial examining the effects of a 10-week progressive resistance training programme were included in this study (17). Participants were recruited from eight National Health Service (NHS) trusts in England, a special education needs school, a University, a primary care organisation in London, national organisations for people with disabilities, and by word of mouth. Young people aged 10–19 years with spastic CP and the ability to walk independently with or without a mobility aid [i.e., Gross Motor Function Classification System (GMFCS) levels I-III] were included in the study. The GMFCS is a five-level classification system (18). Those in GMFCS level I are able to walk and run and climb stairs without assistance. Those in level II are able to walk in most settings but may use a hand-held mobility device indoors or wheeled mobility to travel long distances. Those in level III can walk using a hand-held mobility device but use a wheelchair or powered mobility outdoors. Young people were excluded if they had orthopaedic surgery of the lower limbs in the past 12 months, botulinum toxin type A injections or serial casting in the past 6 months, or insufficient cognition to comply with assessment procedures and the training programme. Approval was obtained from Brunel University London's College of Health, Medicine and Life Sciences Research Ethics Committee and the Surrey Borders Research Ethics Committee (ref: 15/LO/0843). Participants 16 years and over provided written consent. Participants under 16 years provided assent, and parents of participants under 16 years provided written consent.

Assessments were conducted at baseline, 10 and 22 weeks. Demographic and CP-related information was collected at baseline using a standardised questionnaire. Anatomical distribution was described as unilateral or bilateral. Functional mobility was classified according to the GMFCS. Participants selected a statement that best described their mobility based on descriptors of each GMFCS level. Two physiotherapists retrospectively cross-referenced subjective ratings of GMFCS level against video recordings of participants, obtained as part of the baseline assessment. Participants were also asked if they had autism spectrum disorder (ASD), attention deficit hyperactivity disorder (ADHD), behavioural problems, speech problems, epilepsy, or learning difficulties. We combined those with ASD, ADHD or behavioural problems into one category as less than five people reported having ASD and ADHD, respectively.

### Outcomes

The primary outcome in this study was the binary outcome of whether or not an individual used a health service. Secondary outcomes were number of visits, service provider and setting. At each assessment, participants completed a modified Client Service Receipt Inventory (CSRI) to assess health service use (19). At the baseline assessment, participants were asked to state the number of times they visited a range of professionals, number of visits to the emergency department, and number of hospital admissions in the past 12 weeks. They were also asked to state if the service was provided by the NHS, school or privately, and they were asked to state the setting they saw the professional in (i.e., clinic, home or school). At the 10 and 22 week assessment, participants were asked to provide the same information considering the time-period since their last assessment (i.e., the previous 10 and 12 weeks). Assistance was provided by the researcher to read the questions if required. Further, the young person was allowed to ask their parent/guardian or researcher for assistance to answer the questions if required.

### Analysis

The distribution of data was examined using histograms, Q-Q plots, and cross-tabulations. Participant characteristics were described as mean and standard deviation (SD), minimum, maximum, frequencies and percentages, as appropriate. We reported the number and percentage of people with at least one visit to each professional, with at least one visit to the emergency department, and at least one hospital admission. Some participants did not complete the CSRI at 10 or 22 weeks. Therefore, to account for the fact that participants were observed for varying lengths of time, we calculated the incidence rate of at least one visit as the number with at least one visit divided by total person-weeks under observation. We also calculated the rate of visits as total number of visits to each professional divided by total person-weeks. For each service, we examined associations between participant characteristics (i.e., age, sex, GMFCS level, distribution, behavioural problems, speech problems, epilepsy, learning difficulties and type of school) and having at least one visit using separate Cox proportional hazards models. For each service, we also examined associations between participant characteristics and number of visits using separate negative binomial models including an offset for person-time. Where there was evidence that the independent variable was associated with the outcome at the level of α = 10% (i.e., *p* <0.10) in unadjusted analyses, we included these variables together in an adjusted model. Where type of school was not associated with the outcome, we additionally included it in adjusted models as we identified that it confounded the association between a number of participant characteristics and service use. We combined visits to a psychologist and psychiatrist when examining associations because only three people reported visiting a psychiatrist. We did not examine associations with visits to a social worker, chiropodist or audiologist because five or fewer young people reported visiting these professionals. Similarly, we did not examine associations with hospital admissions because less than five children were admitted to hospital. Analyses were performed using Stata version 13.

## Results

Sixty-four participants were recruited to the study. Two participants did not complete the CSRI at any assessment and were therefore excluded from analysis. Sixty-two participants provided data at baseline, 51 participants provided data at 10 weeks, and 50 participants provided data at 22 weeks. Seventy-four percent of participants provided data at all assessments. Participants were observed for a total of 1,854 person-weeks, with a median of 34 weeks per person (min–max: 12–34 weeks). Characteristics of included participants are presented in Table 1. Participants were aged 10–19 years. The majority were male, with bilateral CP, and in mainstream education.

**Table 1:** Participant characteristics.

	***n* (%)**	**Mean (SD); min, max**
Age, yr	62	13.7 (2.5); 10, 19
Female	26 (41.9)	
Male	36 (58.1)	
**GMFCS level**		
I	28 (45.2)	
II	25 (40.3)	
III	9 (14.5)	
**Distribution**		
Unilateral	30 (48.4)	
Bilateral	32 (51.6)	
**Type of school**		
Mainstream education	50 (80.6)	
SEN	12 (19.4)	
**Presence of additional impairment**		
Epilepsy	7 (11.3)	
Speech impairment	14 (22.6)	
Learning difficulties	20 (32.3)	
ASD/ADHD/Behaviour impairment	9 (14.5)	

*ADHD, attention deficit hyperactivity disorder; ASD, autism spectrum disorder; GMFCS, Gross motor function classification system; SD, Standard deviation; SEN, Special education needs*.

### Patterns of Service Use

Participants' use of each service is described in Table 2. Physiotherapists were the most commonly visited professional with 67.7% of participants visiting a physiotherapist at least once, followed by dentists (66.1%), general practitioners (GPs) (48.4%), occupational therapists (40.3%) and orthopaedic surgeons (40.3%). Physiotherapists were also the most frequently visited professional with a total of 473 visits (13.3 visits per person-year). Speech and language therapists (5.0 visits per person-year), occupational therapists (4.5 visits per person-year), and nurses (4.3 visits per person-year) were the next most frequently visited professionals. Nine participants (14.5%) attended the emergency department at least once. The rate of emergency department visits was 0.31 per person-year. Only three participants (4.8%) had a hospital admission, resulting in an admission rate of 0.08 per person-year. For all services except for counselling and social work, the majority of participants accessed the service through the NHS (Table 2). The majority of participants attended a clinic for most services (Table 2). However, many young people received rehabilitation services in school. Thirteen of 25 participants received occupational therapy in school, 12 of 13 participants received speech and language therapy in school, four of seven participants received counselling in school, and 19 of 42 participants received physiotherapy in school.

**Table 2:** Description of visits to each professional, emergency department visits, and hospital admissions.

	**≥1 visit, *n* (%)**	**Incidence rate (95% CI), per person-year**	**Visits, n**	Rate of visits (95% CI^*^), per person-year	**Provider**, ***n***^****a****^				**Setting**, ***n***^****a****^		
					**NHS**	**Private**	**School**	**Other**	**Clinic**	**School**	**Home**
Physiotherapist^e^	42 (67.7)	1.18 (0.87, 1.59)	473	13.27 (8.82, 19.96)	36	4	2	0	25	19	5
Dentist^c, f^	41 (66.1)	1.15 (0.85, 1.56)	84	2.36 (1.82, 3.06)	34	6	0	0	37	1	0
General practitioner^d^	30 (48.4)	0.84 (0.59, 1.20)	74	2.08 (1.41, 3.06)	30	0	0	0	29	1	1
Occupational therapist^d^	25 (40.3)	0.70 (0.47, 1.04)	160	4.49 (2.38, 8.48)	25	2	1	0	10	13	4
Orthopaedic surgeon^e^	25 (40.3)	0.70 (0.47, 1.04)	43	1.21 (0.80, 1.81)	25	1	0	0	22	0	0
Orthotist	24 (38.7)	0.67 (0.45, 1.00)	44	1.23 (0.82, 1.84)	23	1	0	0	18	1	0
Optician^c^^,^ ^g^	24 (38.7)	0.67 (0.45, 1.00)	36	1.01 (0.71, 1.43)	16	7	0	1	22	0	0
Nurse^b^^,^ ^e^	18 (29.0)	0.50 (0.32, 0.80)	152	4.26 (0.89, 20.44)	17	0	0	0	13	4	0
Paediatrician	18 (29.0)	0.50 (0.32, 0.80)	33	0.93 (0.52, 1.65)	18	0	0	0	17	0	0
Speech and language therapist^d^	13 (21.0)	0.36 (0.21, 0.63)	179	5.02 (1.54, 16.35)	13	0	2	0	3	12	0
Other medical specialist^b^	12 (19.4)	0.34 (0.19, 0.59)	17	0.48 (0.28, 0.82)	10	1	0	0	12	0	0
Neurologist	9 (14.5)	0.25 (0.13, 0.49)	14	0.39 (0.18, 0.88)	9	0	0	0	9	0	0
Psychologist^c^^,^ ^g^	8 (12.9)	0.22 (0.11, 0.45)	27	0.76 (0.30, 1.90)	5	0	0	1	4	3	0
Counsellor	7 (11.3)	0.20 (0.09, 0.41)	32	0.90 (0.41, 1.96)	3	0	4	0	3	4	0
Social worker	5 (8.1)	0.14 (0.06, 0.34)	9	0.25 (0.10, 0.63)	2	0	0	3	2	0	4
Chiropodist	4 (6.5)	0.11 (0.04, 0.30)	4	0.11 (0.04, 0.29)	3	1	0	0	3	1	0
Psychiatrist^b^	3 (4.8)	0.08 (0.03, 0.26)	4	0.11 (0.03, 0.36)	2	0	0	0	3	0	0
Audiologist	1 (1.6)	0.03 (0.004, 0.199)	2	0.06 (0.01, 0.40)	0	1	0	0	1	0	0
Emergency department	9 (14.5)	0.25 (0.13, 0.49)	11	0.31 (0.16, 0.59)							
Hospital admission	3 (4.8)	0.08 (0.03, 0.25)	3	0.08 (0.03, 0.25)							

* *Calculated using robust sandwich estimator of variance;*

a *person may select more than one category;*

b *data on provider missing for one person;*

c *data on provider missing for two people;*

d *data on setting missing for one person;*

e *data on setting missing for three people;*

f *data on setting missing for four people;*

g *data on setting missing for two people*.

### Participant Characteristics Associated With at Least One Visit to a Professional

#### Unadjusted Analyses

Unadjusted associations between participant characteristics and having at least one visit to each professional are reported in Tables 3, 4. In unadjusted analyses, there was some evidence that GMFCS level was positively associated with at least one visit to an occupational therapist (*p* = 0.030), speech and language therapist (*p* = 0.022), nurse (*p* = 0.078) and paediatrician (*p* = 0.033). People with bilateral CP were more likely to visit a paediatrician than people with unilateral CP (HR: 3.43, 95% CI 1.13, 10.43; *p* = 0.030). People with ASD/ADHD/behaviour impairment were more likely to visit the emergency department (HR: 3.84, 95% CI 1.03, 14.31; *p* = 0.045). People with epilepsy were more likely to visit an occupational therapist (HR: 2.60, 95% CI 1.04, 6.50; *p* = 0.042) and speech and language therapist (HR 3.70, 95% CI 1.14, 12.00; *p* = 0.030). People with learning difficulties were more likely to visit an occupational therapist (HR: 2.79, 95% CI 1.27, 6.15; *p* = 0.011), speech and language therapist (HR: 3.57, 95% CI 1.17, 10.90; *p* = 0.026), psychologist or psychiatrist (HR: 4.49, 95% CI 1.12, 17.97; *p* = 0.034) and optician (HR: 2.26, 95% CI 1.02, 5.03; *p* = 0.046). People with a speech impairment were more likely to visit a speech and language therapist (HR: 7.70, 95% CI 2.37, 25.04; *p* = 0.001). People attending a SEN school were more likely to visit a speech and language therapist than those attending a mainstream school (HR: 4.19, 95% CI 1.41, 12.46; *p* = 0.010).

**Table 3:** Unadjusted associations with at least one visit to a physiotherapist, occupational therapist, speech and language therapist, psychologist/psychiatrist, orthotist, counsellor, optician.

	**Physiotherapist unadjusted HR (95% CI)**	**Occupational therapist unadjusted HR (95% CI)**	**SALT** **unadjusted HR (95% CI)**	**Psychologist/** **Psychiatrist unadjusted HR (95% CI)**	**Orthotist unadjusted HR (95% CI)**	**Counsellor unadjusted HR (95% CI)**	**Optician unadjusted HR (95% CI)**
Age	0.95 (0.84, 1.08)	1.02 (0.87, 1.20)	0.98 (0.78, 1.22)	0.90 (0.68, 1.19)	0.91 (0.76, 1.08)	1.02 (0.76, 1.37)	1.02 (0.87, 1.19)
**Sex**							
Female	0.83 (0.45, 1.54)	1.31 (0.60, 2.88)	1.40 (0.47, 4.18)	0.98 (0.26, 3.63)	0.87 (0.38, 1.95)	2.98 (0.58, 15.36)	1.20 (0.54, 2.67)
**GMFCS**							
Level II	1.39 (0.71, 2.73)	**2.66 (1.01, 7.00)**	**4.40 (0.91, 21.20)**	1.26 (0.25, 6.24)	1.49 (0.64, 3.45)	2.49 (0.46, 13.59)	1.03 (0.43, 2.49)
Level III	2.04 (0.87, 4.78)	**4.06 (1.31, 12.61)**	**8.50 (1.55, 46.50)**	4.26 (0.86, 21.15)	0.83 (0.18, 3.81)	2.16 (0.20, 23.88)	1.61 (0.51, 5.07)
**Distribution**							
Bilateral	1.18 (0.64, 2.17)	0.90 (0.41, 1.97)	2.20 (0.68, 7.16)	3.45 (0.72, 16.59)	1.16 (0.52, 2.58)	0.39 (0.08, 2.03)	1.39 (0.62, 3.13)
Behaviour impairment	0.67 (0.26, 1.71)	0.94 (0.32, 2.73)	1.45 (0.40, 5.29)	2.46 (0.61, 9.83)	0.70 (0.21, 2.35)	1.92 (0.37, 9.90)	0.96 (0.33, 2.81)
Epilepsy	1.37 (0.58, 3.25)	**2.60 (1.04, 6.50)**	**3.70 (1.14, 12.00)**	1.03 (0.13, 8.24)	0.36 (0.05, 2.65)	3.28 (0.64, 16.90)	1.17 (0.35, 3.92)
Learning difficulties	1.50 (0.81, 2.79)	**2.79 (1.27, 6.15)**	**3.57 (1.17, 10.90)**	**4.49 (1.12, 17.97)**	1.33 (0.58, 3.04)	0.89 (0.17, 4.59)	**2.26 (1.02, 5.03)**
Speech impairment	1.72 (0.91, 3.27)	1.91 (0.84, 4.33)	**7.70 (2.37, 25.04)**	1.74 (0.44, 6.97)	1.15 (0.46, 2.90)	0.58 (0.07, 4.83)	1.19 (0.47, 2.99)
**School**							
SEN	1.30 (0.65, 2.59)	1.71 (0.74, 3.96)	**4.19 (1.41, 12.46)**	2.92 (0.78, 10.88)	0.52 (0.15, 1.74)	2.70 (0.60, 12.06)	1.21 (0.48, 3.04)

*Bold text indicates p < 0.010.*

*CI, confidence interval; GMFCS, Gross motor function classification system; SALT, speech and language therapist; SEN, Special education needs*.

**Table 4:** Unadjusted associations with at least one visit to a general practitioner, nurse, dentist, paediatrician, orthopaedic surgeon, neurologist, other medical specialist and emergency department.

	**GP** **unadjusted HR (95% CI)**	**Nurse** **unadjusted HR (95% CI)**	**Dentist unadjusted HR (95% CI)**	**Paediatrician unadjusted HR (95% CI)**	**Orthopaedic surgeon unadjusted HR (95% CI)**	**Neurologist unadjusted HR (95% CI)**	**Other medical specialist unadjusted HR (95% CI)**	**ED** **unadjusted HR (95% CI)**
Age	0.98 (0.85, 1.14)	1.04 (0.86, 1.24)	0.97 (0.86, 1.10)	0.93 (0.76, 1.13)	0.92 (0.78, 1.08)	0.88 (0.66, 1.17)	0.90 (0.70, 1.15)	0.88 (0.66, 1.17)
**Sex**								
Female	1.05 (0.51, 2.15)	0.96 (0.38, 2.42)	0.86 (0.46, 1.60)	1.51 (0.59, 3.81)	0.69 (0.30, 1.56)	2.42 (0.61, 9.69)	0.40 (0.11, 1.47)	0.96 (0.26, 3.57)
**GMFCS**								
Level II	0.91 (0.40, 2.04)	**2.00 (0.65, 6.12)**	0.76 (0.39, 1.47)	**2.84 (0.87, 9.23)**	1.24 (0.52, 2.98)	0.76 (0.18, 3.16)	1.53 (0.47, 5.02)	0.43 (0.09, 2.11)
Level III	1.90 (0.73, 4.96)	**4.31 (1.25, 14.92)**	0.73 (0.25, 2.11)	**5.34 (1.43, 19.90)**	2.06 (0.70, 6.05)	0.88 (0.10, 7.52)	0.90 (0.10, 7.67)	0.74 (0.09, 6.15)
**Distribution**								
Bilateral	1.49 (0.72, 3.09)	1.55 (0.60, 3.99)	0.77 (0.41, 1.42)	**3.43 (1.13, 10.43)**	1.25 (0.57, 2.75)	3.48 (0.72, 16.73)	1.99 (0.60, 6.60)	1.24 (0.33, 4.61)
Behaviour impairment	0.74 (0.26, 2.12)	9.96 (0.28, 3.33)	1.08 (0.45, 2.27)	0.97 (0.28, 3.35)	0.94 (0.32, 2.75)	1.39 (0.29, 6.69)	0.96 (0.21, 4.37)	**3.84 (1.03, 14.31)**
Epilepsy	1.27 (0.44, 3.63)	2.35 (0.77, 7.15)	1.14 (0.45, 2.90)	*	1.12 (0.33, 3.74)	2.33 (0.48, 11.22)	*	1.04 (0.13, 8.28)
Learning difficulties	1.31 (0.62, 2.76)	**2.23 (0.88, 4.62)**	1.28 (0.68, 2.43)	1.79 (0.71, 4.53)	1.25 (0.55, 2.83)	1.81 (0.49, 6.75)	1.62 (0.51, 5.11)	1.81 (0.49, 6.75)
Speech impairment	1.77 (0.83, 3.78)	2.21 (0.86, 5.70)	0.98 (0.47, 2.05)	1.71 (0.64, 4.56)	1.35 (0.56, 3.23)	1.79 (0.45, 7.16)	1.76 (0.53, 5.86)	1.00 (0.21, 4.82)
**School**								
SEN	1.10 (0.47, 2.56)	1.38 (0.49, 3.88)	0.75 (0.33, 1.69)	1.38 (0.49, 3.87)	0.92 (0.34, 2.45)	1.04 (0.22, 5.03)	1.20 (0.32, 4.43)	0.45 (0.06, 3.59)

*Bold text indicates p < 0.010.*

*Unable to calculate.

*CI, confidence interval; ED, Emergency department; GMFCS, Gross motor function classification system; GP, General practitioner; SEN, Special education needs*.

#### Adjusted Analyses

As presented in Table 5, in adjusted analyses, people in GMFCS level III remained more likely to visit an occupational therapist (adjusted HR: 4.23, 95% CI 1.25, 14.30; *p* = 0.020) and nurse (adjusted HR: 4.32, 95% CI 1.17, 16.00; *p* = 0.028) than those in level I. There was also weak evidence that people with learning difficulties were more likely to visit an occupational therapist (adjusted HR: 2.35, 95% CI 1.00, 5.48; *p* = 0.049) and people with a speech impairment were more likely to visit a speech and language therapist (adjusted HR: 4.14, 95% CI 0.97, 17.62; *p* = 0.055).

**Table 5:** Adjusted associations with at least one visit to an occupational therapist, speech and language therapist, nurse, optician, paediatrician.

	**Occupational therapy adjusted HR (95% CI); *p*-value**	**SALT** **adjusted HR (95% CI);** ***p*-value**	**Nurse** **adjusted HR (95% CI);** ***p*-value**	**Paediatrician** **adjusted HR (95% CI);** ***p*-value**
**GMFCS**				
Level II	2.18 (0.80, 5.92); 0.126	2.40 (0.43, 13.31); 0.315	1.93 (0.63, 5.93); 0.253	2.14 (0.61, 7.50); 0.234
Level III	4.23 (1.25, 14.30); 0.020	4.54 (0.56, 36.57); 0.155	4.32 (1.17, 16.00); 0.028	3.05 (0.65, 13.23); 0.156
**Distribution**				
Bilateral	–	–	–	2.33 (0.66, 8.18); 0.188
Behaviour impairment	–	–	–	–
Epilepsy	2.12 (0.73, 6.15); 0.165	2.09 (0.35, 12.63); 0.420	–	
Learning difficulties	2.35 (1.00, 5.48); 0.049	1.97 (0.59, 6.53); 0.270	2.24 (0.80, 6.24); 0.123	–
Speech impairment	–	4.14 (0.97, 17.62); 0.055	–	–
**School**				
SEN	0.77 (0.30, 1.99); 0.585	0.84 (0.15, 4.75); 0.847	0.71 (0.21, 2.36); 0.574	1.17 (0.39, 3.50); 0.776

*CI, confidence interval; GMFCS, Gross motor function classification system; SALT, speech and language therapist; SEN, Special education needs*.

### Participant Characteristics Associated With Number of Visits to a Professional

#### Unadjusted Analyses

Unadjusted associations between participant characteristics and number of visits to each professional are reported in Tables 6, 7. Age was negatively associated with visits to a GP (IRR: 0.84, 95% CI 0.72, 0.97; *p* = 0.019) and positively associated with visits to a nurse (IRR: 1.58, 95% CI 1.13, 2.22; *p* = 0.008). Compared to people in GMFCS level I, people in GMFCS level II had more visits to a physiotherapist (IRR: 2.80, 95% CI 1.17, 6.68; *p* = 0.020), occupational therapist (IRR: 4.99, 95% CI 1.48, 16.80; *p* = 0.009), speech and language therapist (IRR: 31.52 (95% CI 4.69, 211.97; *p* < 0.001), nurse (IRR: 24.64, 95% CI 5.11, 118.80; *p* < 0.001), dentist (IRR: 0.45, 95% CI 0.26, 0.79; *p* = 0.005) and paediatrician (IRR: 3.71, 95% CI 1.12, 12.26; *p* = 0.032). People in GMFCS level III also had more visits to a paediatrician than people in level I (IRR: 7.70, 95% CI 1.94, 30.50; *p* = 0.004). People with bilateral CP had more visits to a physiotherapist (IRR: 3.15, 95% CI 1.42, 6.99; *p* = 0.005), an occupational therapist (IRR 3.82, 95% CI 1.22, 11.92; *p* = 0.021), and a paediatrician (IRR: 3.95, 95% CI 1.32, 11.83; *p* = 0.014) and less visits to a nurse (IRR: 0.17, 95% CI 0.04, 0.78; *p* = 0.022) than those with unilateral CP. People with epilepsy had more visits to a speech and language therapist (IRR: 72.61, 95% CI 16.69, 315.89; *p* < 0.001), nurse (IRR: 36.97, 95% CI 7.19, 189.99; *p* < 0.001) and neurologist (IRR: 5.58, 95% CI 1.12, 27.71; *p* = 0.035). People with learning difficulties had more visits to an occupational therapist (IRR: 4.25, 95% CI 1.32, 13.62; *p* = 0.015), psychologist or psychiatrist (IRR: 7.60, 95% CI 1.38, 41.99; *p* = 0.020), and nurse (IRR: 14.71, 95% CI 3.43, 61.13, *p* < 0.001). People with a speech impairment had more visits to a physiotherapist (IRR: 3.48, 95% CI 1.38, 8.78; *p* = 0.008), speech and language therapist (IRR: 72.61, 95% CI 16.69, 315.89; *p* < 0.001), GP (IRR: 2.24, 95% CI 1.02, 4.89; *p* = 0.044), and nurse (IRR: 21.01, 95% CI 4.93, 89.60; *p* < 0.001). People attending a special educational needs school had more visits to a speech and language therapist (IRR: 19.03, 95% CI 2.40, 145.16; *p* = 0.004) and nurse (IRR: 19.52, 95% CI 4.16, 91.54; *p* < 0.001) than those in mainstream education.

**Table 6:** Unadjusted associations with number of visits to a physiotherapist, occupational therapist, speech and language therapist, psychologist/psychiatrist, orthotist, counsellor, optician.

	**Physiotherapist unadjusted IRR (95% CI)**	**Occupational therapist unadjusted IRR (95% CI)**	**SALT** **unadjusted IRR (95% CI)**	**Psychologist/** **Psychiatrist unadjusted IRR (95% CI)**	**Orthotist unadjusted IRR (95% CI)**	**Counsellor unadjusted IRR (95% CI)**	**Optician unadjusted IRR (95% CI)**
Age	**0.85 (0.71, 1.01)**	0.89 (0.70, 1.14)	0.74 (0.44, 1.26)	0.72 (0.44, 1.19)	**0.87 (0.73, 1.03)**	0.95 (0.63, 1.45)	1.03 (0.90, 1.18)
**Sex**							
Female	1.11 (0.47, 2.59)	2.64 (0.73, 9.51)	3.44 (0.47, 24.99)	2.14 (0.34, 13.56)	1.08 (0.47, 2.49)	2.39 (0.26, 21.89)	1.02 (0.50, 2.05)
**GMFCS**							
Level II	**2.80 (1.17, 6.68)**	**4.99 (1.48, 16.80)**	**31.5 (4.7, 212.0)**	0.78 (0.11, 5.75)	1.87 (0.81, 4.29)	1.47 (0.14, 15.08)	0.93 (0.44, 1.99)
Level III	**3.22 (0.97, 10.66)**	**4.33 (0.82, 22.75)**	**5.4 (0.4, 72.8)**	0.61 (0.03, 10.72)	0.43 (0.08, 2.25)	0.24 (0.01, 9.74)	0.99 (0.34, 2.88)
**Distribution**							
Bilateral	**3.15 (1.42, 6.99)**	**3.82 (1.22, 11.92)**	0.47 (0.06, 3.45)	1.10 (0.17, 7.04)	0.96 (0.42, 2.21)	0.25 (0.03, 2.18)	1.17 (0.58, 2.35)
Behaviour impairment	0.72 (0.22, 2.38)	2.57 (0.50, 13.11)	1.8 (0.1, 31.3)	6.22 (0.68, 56.61)	0.67 (0.20, 2.29)	4.12 (0.22, 76.47)	1.28 (0.53, 3.12)
Epilepsy	1.38 (0.37, 5.19)	**5.28 (0.96, 29.04)**	**26.5 (2.3, 306.6)**	3.94 (0.28, 55.60)	0.98 (0.27, 3.61)	4.34 (0.17, 111.7)	1.03 (0.34, 3.15)
Learning difficulties	2.01 (0.84, 4.83)	**4.25 (1.32, 13.62)**	1.04 (0.12, 9.12)	**7.60 (1.38, 41.99)**	1.80 (0.79, 4.11)	1.50 (0.13, 15.98)	1.67 (0.84, 3.34)
Speech impairment	**3.48 (1.38, 8.78)**	**3.62 (0.96, 13.62)**	**72.6 (16.7, 315.9)**	0.66 (0.07, 6.28)	1.21 (0.47 (3.13)	0.46 (0.03, 7.03)	0.93 (0.40, 2.15)
**School**							
SEN	1.72 (0.61, 4.90)	3.08 (0.74, 12.83)	19.0 (2.5, 145.2)	1.39 (0.14, 13.99)	0.82 (0.29, 2.36)	2.92 (0.20, 42.74)	1.27 (0.56, 2.85)

*Bold text indicates *p* < 0.010.*

*CI, confidence interval; GMFCS, Gross motor function classification system; SALT, speech and language therapist; SEN, Special education needs.*

**Table 7:** Unadjusted associations with number of visits to a general practitioner, nurse, dentist, paediatrician, orthopaedic surgeon, neurologist, other medical specialist and emergency department.

	**GP** **unadjusted IRR (95% CI)**	**Nurse** **unadjusted IRR (95% CI)**	**Dentist unadjusted IRR (95% CI)**	**Paediatrician unadjusted IRR (95% CI)**	**Orthopaedic surgeon unadjusted IRR (95% CI)**	**Neurologist unadjusted IRR (95% CI)**	**Other medical specialist unadjusted IRR (95% CI)**	**ED** **unadjusted IRR (95% CI)**
Age	**0.84 (0.72, 0.97)**	**1.58 (1.13, 2.22)**	0.98 (0.88, 1.09)	0.92 (0.74, 1.14)	0.89 (0.74, 1.07)	0.80 (0.54, 1.19)	0.87 (0.68, 1.11)	0.88 (0.66, 1.18)
**Sex**								
Female	1.35 (0.64, 2.81)	**0.11 (0.02, 0.53)**	0.71 (0.42, 1.20)	2.30 (0.82, 6.41)	1.01 (0.44, 2.31)	2.98 (0.67, 13.18)	0.53 (0.16, 1.75)	1.08 (0.29, 3.95)
**GMFCS**								
Level II	1.24 (0.56, 2.73)	**24.6 (5.1, 118.8)**	**0.45 (0.26, 0.79)**	**3.71 (1.12, 12.26)**	1.16 (0.47, 2.82)	0.48 (0.10, 2.36)	0.97 (0.29, 3.26)	0.29 (0.06, 1.42)
Level III	1.12 (0.36, 3.44)	**3.4 (0.4, 30.7)**	**0.64 (0.30, 1.36)**	**7.70 (1.94, 30.50)**	1.34 (0.40, 4.50)	0.35 (0.02, 4.54)	0.81 (0.13, 4.95)	0.43 (0.05, 3.72)
**Distribution**								
Bilateral	**1.85 (0.89, 3.85)**	**0.17 (0.04, 0.78)**	0.91 (0.54, 1.53)	**3.95 (1.32, 11.83)**	1.24 (0.54, 2.83)	1.32 (0.30, 5.87)	1.30 (0.42, 4.06)	0.79 (0.22, 2.88)
Behaviour impairment	1.15 (0.42, 3.17)	0.17 (0.02, 1.90)	1.51 (0.79, 2.87)	0.53 (0.11, 2.64)	1.72 (0.62, 4.74)	3.87 (0.77, 19.53)	1.14 (0.25, 5.18)	3.03 (0.80, 11.45)
Epilepsy	**2.62 (0.99, 6.95)**	**37.0 (7.2, 190.0)**	0.62 (0.24, 1.61)	*	1.43 (0.43, 4.67)	**5.58 (1.12, 27.71)**	*	0.80 (0.09, 7.37)
Learning difficulties	1.72 (0.82, 3.64)	**14.7 (3.5, 61.1)**	0.97 (0.55, 1.69)	1.88 (0.66, 5.36)	1.41 (0.61, 3.26)	2.56 (0.60, 11.00)	1.42 (0.45, 4.50)	1.18 (0.31, 4.51)
Speech impairment	**2.24 (1.02, 4.89)**	**21.0 (4.9, 89.6)**	0.76 (0.40, 1.45)	2.15 (0.70, 6.61)	0.62 (0.22, 1.75)	0.86 (0.14, 5.10)	1.34 (0.38, 4.74)	0.74 (0.14, 3.87)
**School**								
SEN	1.77 (0.75, 4.17)	**19.5 (4.2, 91.5)**	0.68 (0.34, 1.36)	2.15 (0.67, 6.91)	0.60 (0.20, 1.81)	0.63 (0.09, 4.55)	1.57 (0.44, 5.66)	0.38 (0.05, 3.25)

*Bold text indicates p < 0.010.*

*Unable to calculate.

*CI, confidence interval; ED, Emergency department; GMFCS, Gross motor function classification system; GP, General practitioner; SEN, Special education needs*.

#### Adjusted Analyses

Results from adjusted analyses are reported in Table 8. In adjusted analyses, people with learning difficulties had more visits to an occupational therapist (adjusted IRR: 5.6, 95% CI 1.7, 18.0; *p* = 0.004). People with a speech impairment (adjusted IRR: 19.5, 95% CI 3.9, 97.7; < 0.001) and epilepsy (IRR: 6.3, 95% CI 1.2, 34.4; *p* = 0.032) had more visits to a speech and language therapist. Age remained negatively associated with rate of GP visits (adjusted IRR: 0.85, 95% CI 0.74, 0.99; *p* = 0.035). People with epilepsy had more visits to a nurse (adjusted IRR: 18.1, 95% CI 2.0, 167.0; *p* = 0.010) and people in GMFCS level II had more visits to a nurse than people in GMFCS level I (adjusted IRR: 5.4, 95% CI 1.1, 26.6; *p* = 0.040).

**Table 8:** Adjusted associations with number of visits to a physiotherapist, occupational therapist, speech and language therapist, general practitioner, nurse, paediatrician.

	**Physiotherapist adjusted IRR (95% CI); *p*-value**	**Occupational therapist** **adjusted IRR (95% CI); *p*-value**	**SALT** **adjusted IRR (95% CI); *p*-value**	**GP** **adjusted IRR (95% CI); *p*-value**	**Nurse** **adjusted IRR (95% CI); *p*-value**	**Paediatrician adjusted IRR (95% CI); *p*-value**
Age	0.82 (0.66, 1.01); 0.067	–	–	0.85 (0.74, 0.99); 0.035	1.05 (0.77, 1.42); 0.760	
**Sex**						
Female	–	–	–	–	0.24 (0.06, 0.96); 0.043	
GMFCS						
Level II	1.57 (0.61, 4.05); 0.353	3.3 (0.8, 14.7); 0.113	1.9 (0.3, 11.3); 0.481	–	5.4 (1.1, 26.6); 0.040	2.92 (0.86, 9.90); 0.086
Level III	2.23 (0.56, 8.85); 0.254	7.2 (0.9, 56.9); 0.060	0.9 (0.1, 10.3); 0.926	–	8.8 (0.5, 142.6); 0.124	3.9 (0.8, 19.5); 0.104
**Distribution**						
Bilateral	1.64 (0.65, 4.15); 0.298	0.81 (0.17, 3.78); 0.787	–	1.69 (0.84, 3.40); 0.145	0.95 (0.16, 5.54); 0.950	2.45 (0.75, 8.04); 0.140
Behaviour impairment	–	–	–	–	–	
Epilepsy	–	3.8 (0.6, 22.3); 0.142	6.3 (1.2, 34.4); 0.032	2.08 (0.76, 5.66); 0.153	18.1 (2.0, 167.0); 0.010	
Learning difficulties	–	5.6 (1.7, 18.0); 0.004	–	–	1.53 (0.35, 6.63); 0.568	
Speech impairment	2.38 (0.87, 6.46); 0.090	2.43 (0.60, 9.91); 0.215	19.5 (3.9, 97.7); <0.001	1.38 (0.58, 3.28); 0.465	1.8 (0.3, 10.7); 0.493	
**School**						
SEN	1.31 (0.41, 4.26); 0.649	0.75 (0.17, 3.24); 0.697	3.0 (0.7, 13.8); 0.156	1.21 (0.45, 3.30); 0.703	0.44 (0.05, 3.63); 0.443	1.45 (0.44, 4.80); 0.546

*CI, confidence interval; GMFCS, Gross motor function classification system; GP, General practitioner; SALT, speech and language therapist; SEN, Special education needs*.

## Discussion

We aimed to describe patterns of visits to rehabilitation professionals, medical professionals, emergency department visits and hospital admissions among ambulatory young people with CP living in England, and identify factors associated with service use. In our sample, physiotherapists were the most commonly visited professional, with 68% of young people visiting a physiotherapist at least once, and also the most frequently visited professional, with a visit rate of 13.3 per person-year. A similar proportion of young people visited a dentist at least once. All other professions were visited by less than half of participants during the study period. Despite only 21% of young people visiting a speech and language therapist and only 40% visiting on occupational therapist, these were the second and third most frequently visited professionals, with visit rates of 5.0 and 4.5 per person-year, respectively. In adjusted analyses, GMFCS level, age and presence of epilepsy, learning difficulties and speech impairment were associated with having at least one visit and number of visits to a range of professionals.

Few studies that report service use among young people with CP have examined the range of services examined in this study. Estimates for the proportion of young people visiting an occupational therapist (44%) and speech and language therapist (19%) in the Netherlands (4) were almost identical to estimates from this study. The proportion of young people visiting a physiotherapist was higher than that reported among 13–18 year olds in the United States (59.4% over 12 months) (13) and 12–19 year olds in the Netherlands (51.8% over 6 months) (4). In contrast, the proportion visiting a physiotherapist, occupational therapist and speech and language therapist was lower in this study than in a study of young people aged 12–18 years in GMFCS levels IV and V in Northern Ireland (92, 62, and 39%, respectively) (5).

Less than half of young people visited a medical professional during the study period. The proportion visiting an orthopaedic surgeon, at 40%, was lower than that reported among young people in GMFCS levels IV and V in Northern Ireland (64% over 6 months) (5). While the difference may be partly explained by difference in GMFCS level, this study also excluded individuals who had received orthopaedic surgery of the lower limbs in the past year, which may have resulted in a biased sample. A third of ambulatory young people in France visited a neurologist (20), which is much higher than the 14.5% of our sample. The higher proportion of people visiting a neurologist in France may partly be explained by a higher prevalence of epilepsy in the sample. Compared to other professions, the number of young people visiting a psychologist or psychiatrist was small. Only 5% of young people in this study visited a psychiatrist, which is identical to the proportion of ambulatory young people who visited a psychiatrist in France (20). Thirteen percent visited a psychologist, which is similar to the 15% who visited a psychologist in the study in the Netherlands (4) but higher than the 3% reported in the study in Northern Ireland (5).

The relatively small number of people visiting a paediatrician or neurologist suggests that a medical professional is not co-ordinating their care. Only 15% of young people with CP in the UK reported having a person they can easily contact to support with co-ordination of care (21). Further, in the UK, nearly half of young people with CP are discharged from paediatric services to their GP, in the absence of dedicated adult services (11). In this study, the rate of visits to GPs, at approximately 2 per person year, is nearly identical to that reported from a recent analysis of primary care data of children aged 10–14 years in England (12). The same analysis indicated that the rate of visits increased to approximately 2.5 per person year at age 15–19 years and 3.5 per person year at 20–24 years (12), which corresponds with the GP becoming the co-ordinator of care. We however, found that the rate of visits to GPs declined with age. This may be because we included those in GMFCS levels I-III, who potentially do not report health issues until adulthood, and thus there may be a delay between being discharged from paediatric services and requiring access to adult services via their GP. A study in France similarly observed that the proportion of ambulatory young people visiting a GP was lower in those aged 12–17 years compared to those aged 6–11 years but increased in the group aged 18 years and older (20). This potential decline in visits to a GP in adolescence may result in the GP being unfamiliar with the young person's medical history and contribute to the lack of continuity of care reported by young adults with CP (6).

We found limited data on service use among young people with other long-term conditions in the UK or internationally. A report from the UK Cystic Fibrosis Registry indicated that 96% of people with cystic fibrosis received an annual review in 2019 (22). Seventy-eight percent of people with cystic fibrosis under 18 received any form of positive expiratory pressure and 66% received an exercise intervention from a physiotherapist (22). Among 175 children with attention deficit/hyperactivity disorder (ADHD) in the UK, use of medical services was higher compared to that in our study, with 59% visiting a hospital, 69% visiting a doctor, and 47% visiting a psychologist in the past 6 months (23). Not unlike our findings, a study of 122 young people aged 12–17 years with autism spectrum disorder in Germany found that the dentist/orthodontist was the most commonly visited professional over 12 months (24). However, unlike our study, the paediatrician was the second most commonly visited professional, with 50% visiting the paediatrician at least once in the past year. Nearly 30% of young people also reported visiting a ASD outpatient clinic in the past year. The higher proportion of young people visiting specialist services may explain why only 27% visited the GP in the past year (24), in contrast to 48% of our sample. Conversely, use of physiotherapy, speech and language therapy, and occupational therapy was higher among young people with CP than ASD (24), which concurs with a study that directly compared therapy use between children with CP and ASD in the United States (25).

In unadjusted analyses, we found that GMFCS level was positively associated with having at least one visit to an occupational therapist and speech and language therapist, and having more visits to a physiotherapist and occupational therapist. This is consistent with previous studies that found severity of motor impairment was positively associated with using physiotherapy, occupational therapy, and speech and language therapy (4, 13). However, in adjusted analyses GMFCS level was only associated with visiting an occupational therapist and nurse. In contrast to a previous study (4), we did not find that children in specialist education settings were more likely to receive services. However, this may be because a relatively low proportion of people in this study attended a special educational needs school. In agreement with a study from the Netherlands (4), unadjusted analyses indicated that young people with learning difficulties were more likely to visit a psychologist or psychiatrist. However, in our adjusted analyses, learning difficulties were only associated with visiting an occupational therapist, and not a psychologist or psychiatrist.

While this study shows the proportion of young people with CP using services is generally low, it does not indicate the needs of young people or unmet need. However, some findings do align with needs reported by a group of young people with CP from the UK, the majority of whom were in GMFCS levels I-III (11). Twenty-two percent of young people reported needs relating to speech (11), 42% report needs relating to bone and joint problems, and 45% report needs relating to eyesight. In this study 21% of young people used speech and language therapy, 40% visited an orthopaedic surgeon, and 38% visited an optician. Although the findings may be used to support future service planning, a significant proportion of ambulatory young people with CP in the UK report unmet health needs, which also need to be considered when allocating resources (11). In particular, between 35 and 60% of young people and parents report that needs are not being met in relation to bone and joint problems, pain, and speech (11). There is also a trend toward an increase in unmet health needs with age, highlighting that people with CP continue to require access to services as they transition to adulthood (11).

Understanding existing service use and factors associated with service use is pertinent in order to identify gaps in health care, and to develop services. Despite the relatively high incidence of CP compared to other childhood-onset conditions, there is limited information regarding service use among young people with CP in the NHS. The breadth of health services accessed by young people with CP demonstrates the complexity of the condition and the need for a co-ordinated approach to developing services. In particular, it highlights the lack of condition-specific specialist services available for young people with CP. Combining this data with currently available and future data on the unmet needs of young people with CP is warranted to inform service development that meets the needs of people with CP throughout their life.

A limitation of this study is that participants were recruited largely through the physiotherapists in the NHS, hence potentially biasing the sample toward individuals who visit physiotherapists frequently. However, not all participants were recruited through the NHS. Further, participants recruited through the NHS received information about the study even if they were not currently attending physiotherapy. Importantly our study has a cross-sectional design and therefore we cannot make inferences regarding the change in service use among young people with CP over time. Findings are also limited to young people with spastic CP in GMFCS levels I-III without severe intellectual disability. For context, ~92% of people have spastic CP and ~75% are in GMFCS levels I-III in the UK (16). As the data for this study were collected as part of a trial examining the effects of resistance training, young people were excluded if they had orthopaedic surgery of the lower limbs in the past 12 months, botulinum toxin type A injections or serial casting in the past 6 months. Individuals in receipt of these interventions are likely more frequent users of health services than individuals included in our study, and therefore service use in our sample may be an underestimation of use in the population. Finally, we may not have found associations between participant characteristics and service use because the number of people who visited some services was relatively small.

Despite the relatively high prevalence of CP, there is a stark lack of data on health service use. Data on patterns of use may be helpful for planning future services. Generally the proportion of young people with CP using services was low. In the context of limited resources, data on service use in combination with data on unmet health needs, may support the reorganisation of services to maximise benefits to young people with CP.

## Data Availability Statement

The datasets presented in this article are not readily available because the datasets generated during and/or analysed during the current study are not expected to be made available publicly, as consent was not obtained to publish the anonymised data. Requests to access the datasets should be directed to Jennifer Ryan, jenniferryan@rcsi.com.

## Ethics Statement

Approval was obtained from Brunel University London's College of Health and Life Sciences Research Ethics Committee and the Surrey Borders Research Ethics Committee (ref: 15/LO/0843). Participants 16 years and over provided written consent. Participants under 16 years provided assent, and parents of participants under 16 years provided written consent.

## Author Contributions

JR, CK, and GL conceived and designed the analysis. JR, GL, NT, and MN collected the data. JR and GL performed the analysis and drafted the manuscript. NT, CK, and MN provided critical feedback and contributed to the final manuscript. All authors contributed to the article and approved the submitted version.

## Conflict of Interest

The authors declare that the research was conducted in the absence of any commercial or financial relationships that could be construed as a potential conflict of interest.
